# Voting on Embryonic Stem Cell Research: Citizens More Supportive than Politicians

**DOI:** 10.1371/journal.pone.0170656

**Published:** 2017-01-26

**Authors:** David Stadelmann, Benno Torgler

**Affiliations:** 1 University of Bayreuth, Bayreuth, Germany; 2 Queensland University of Technology, Brisbane, Australia; Faculty of Biochemistry, Biophysics and Biotechnology, Jagiellonian University, POLAND

## Abstract

As the public debate over stem cell research continues, the observable voting behaviour in Switzerland offers a unique opportunity to compare the voting behaviour of politicians with that of voters. By analysing the outcomes of a referendum on a liberal new bill regulating such research, we reveal an about 10 percentage point lower conditional probability of the bill being accepted by politicians than by voters. Whereas the behaviour of politicians is driven almost entirely by party affiliation, citizen votes are driven not only by party attachment but also by church attendance. Seldom or never attending church increases the probability of bill acceptance by over 15 percentage points, while supporting the Liberal Party and the Social Democratic Party instead of the Christian Democratic Party makes supporting the bill more likely for voters, suggesting that religious observance is important. The observance of these tendencies in Switzerland—an environment that promotes discussion through direct democratic rights—strongly suggests that citizens see the benefits of stem cell research.

## Introduction

Although stem-cell research has experienced an explosion of activity since the 1998 isolation of human embryonic stem cells [1], such research has been accompanied by a heated and bitter public debate around which the media have structured their coverage of the issue [2]. A primary trigger for this ongoing controversy was U.S. President George W. Bush’s 2001 national TV appearance announcing a new policy restricting stem cell research [3], which led to legal uncertainties that have affected its use in the U.S. [4]. [5] provide an overview of the stages of scientific, policy, and political development of the stem cell controversy that allows to understand the evolution of media coverage over a prolonged period pointing out the fact that the stem cell controversy coincided with President Bush’s first six months of office “setting the stage for familiar themes revolving around the implementation of campaign promises to influential supporters, anticipation of the president’s first big political test in office, and the president grappling with moral dilemmas that accompany the burden of power” (p. 45). They stress that policy context mattered. In addition, in the case of stem cell research the media storytelling is attractive as it can be framed in terms of “political strategy/conflict and ethics/morality” (p. 66). President Bush also vetoed twice bills that the Congress passed to overturn the restrictions [6]. Nevertheless, the U.S. has been leading human embryonic stem cell research since 1998, with scientists performing well despite restrictive Bush policies [7,8]. In fact, since 2005, U.S. research on derivations has rebounded in spite of rapid progress by other countries like China, Israel, and Singapore [7]. Even the 2001 U.S. federal funding constraints have had no significant impact because the research has shifted geographically into states and countries with more favourable regimes and funding [8]. Nevertheless, stem cell research was hotly debated during the 2006 and 2008 U.S. elections and became a prominent campaign topic across politically strategic states [9]. In fact, several leading researchers have criticized the White House Domestic Policy Council report *Advancing Stem Cell Science Without Destroying Human Life* on the grounds that it misrepresented their work in an attempt to influence the cell debate in Congress [10]. A survey of U.S. stem cell scientists shows that frequency policy changes and ongoing policy uncertainty are the reasons that affect type or quality of the science conducted. Delaying plans to begin human embryonic stem cell research or developing new projects, impeding ongoing research, limiting future funding opportunities, transitioning away or reducing that kind of research, disrupting long-term planning, adopting suboptimal research design, disrupting collaborations, and even considering relocation are key factors that were mentioned due to ongoing policy uncertainty [6].

## Literature and Public Discussion

The debate on stem cell research has predominantly been framed as a moral matter. Opponents stress that embryos are human life and scientists should not be allowed to play God, while proponents emphasize the societal and therapeutic benefits of stem cell research [9]. Such benefits range from transplants to cell replacement therapies that treat such debilitating diseases as diabetes, Parkinson’s, and Huntington’s, research areas that have opened up a new terrain of basic biology [11]. Nonetheless, scientists considering a career in embryonic stem cell biology have been warned that they will face uncertainty and sustainability issues within this touchy research field, in addition to the vigorous and extended public debate between supporters who sensationalize the research and opponents who demonize it [12].

The intense reporting of this debate has to date been more descriptive than empirical, with the political process revealing the field’s ongoing vulnerability [13], which has prompted stem cell researchers to voice concerns about the difficulty of predicting where the political debate will go next [14]. Some even expected the debate to disappear after the use of human embryos through direct cell reprogramming was declared safe for use in patients and new opportunities were created by the development of pluripotent (iPS) cells from individual skin cells. However, the therapies for heart, neurological, and other diseases still pose huge challenges [14]. Nevertheless, since the 2004 transplant of such cells into a woman with eye disease, hopes attached to the use of iPS cells to repair damaged or diseased tissues have been increasing [15]. Today, such iPS cell usage is seen as a new route to research implementing human embryonic stem cells [16], and a recent survey of 26 hospital patients indicated a generally positive and supportive attitude towards donation of biological material for iPS research [17].

Now, therefore, the field is well past the Bush era and in what its leading scientists refer to as a turning point or renaissance [12]. Nevertheless, a new debate has recently emerged over embryo gene editing after some researchers expressed concern that it could be a slippery slope towards unethical or unsafe non-medical uses. Others counter-argued that its application to human embryos could answer basic scientific questions beyond clinical functions [18], a claim that has raised new policy concerns [19]. Without doubt, the moral and ethical dimensions of the controversy suggest that it will not disappear any time soon, meaning that the actions and opinions of all parties involved should be investigated to better understand the debate. Yet the existing empirical literature still relies heavily on studying general public attitudes [9] rather than the *actual behaviour* of individuals. For example, one common attitudinal question asks how much the respondent is in favour of or opposed to medical research that uses stem cells from human embryos ([9], p. 4).

One answer to this query was expressed in the U.S. state of California by a 2004 citizen vote to establish the California Institute for Regenerative Medicine (CIRM), which, with an endowment of $3 billion, is the largest funder of stem cell work in the world. After that vote, five other states set up stem-cell research agencies [20]. In 2017, California voters will again decide whether or not to support CIRM. Meanwhile, the European public’s perception of stem cell research has been expressed in a series of citizens’ initiatives that drew more than 1 million signatures and thus required a formal public hearing in the European Parliament. One petition signed by 1.7 million people requested a ban on financing any activity that required the destruction of human embryos [21].

## Data and Method

Switzerland, particularly, offers an interesting opportunity to study how acceptable stem cell research is to voters and politicians not only because the policy issues decided by parliament are frequently presented to citizens in referenda—whose outcomes are binding and lead to direct policy outcomes—but because parliamentary representatives’ votes are publicly accessible. That is, all final votes in the National Council (Lower House of Parliament; comparable to the U.S. House of Representatives) are carried out through an electronic voting system, and the parliamentary services make public all individual votes registered by the system. Individual votes can thus be compared with citizen votes for or against the status quo on identical legislative proposals [22]. The Council of States (Upper House of Parliament; comparable to the U.S. Senate), in contrast, has no electronic voting system and did not even introduce camera recording until the winter of 2006 [23].

**Fig 1:**
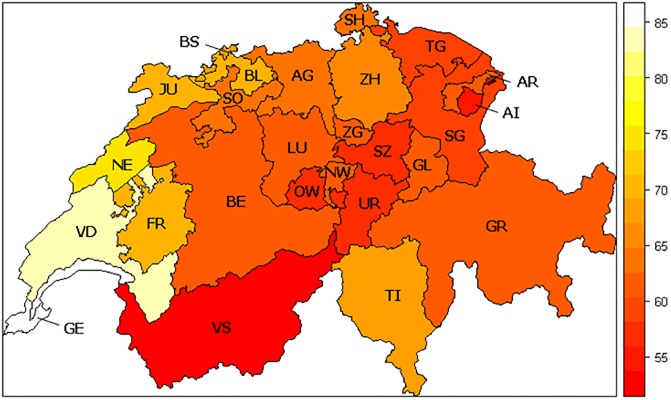
Acceptance rate of the proposed law among all 26 Swiss cantons. Cantons in central Switzerland were less likely to approve the proposal, while the French and Italian speaking cantons were more likely to approve it. Source: Federal Statistical Office and Swissvotes Dataset.

In 2004, Switzerland held a referendum on whether to accept a liberal new bill regulating stem cell research, which was proposed by the Federal Council and a parliamentary majority. An opposing committee was against the new bill and in favour of a ban on embryonic stem cell research. We therefore compare the individual votes on the stem cell research legislation of 160 National Council members with the responses from a representative exit poll sample collected by Vox, which has collated post-survey data after each federal vote since 1977 (for more details, see http://forsdata.unil.ch/projects/voxit/ or [24]). The overall voting outcome reveals substantial heterogeneity among the Swiss cantons even though all accepted the new liberal bill (Fig 1).

Our main dependent variable measures whether individuals (either the National Council members or the citizens polled) accepted the proposed legislation on stem cell research. We perform a logit analysis of the probability to accept the proposal. Descriptive statistics and descriptions for all covariates are given in appendix S1 Table.

## Empirical Results

The analytical results, reported in Table 1, reveal that politicians were less likely than citizens to accept the bill, indicating that the general population was more open to embryonic stem cell research than its representatives. Specifically, being a member of the National Council reduced the probability of favouring the new law by 12.2 percentage points holding constant gender and age as well as characteristics of constituencies; however, the individual characteristics of age, marital status, education, and Roman Catholic faith were unimportant in the decisions of both politicians and voters, although women were more likely to be against the new bill. All our estimates include constituency fixed effects to take account of the possibility that the structure of the economy or the social fabric may be different across constituencies, i.e. certain constituencies may have more clusters of research institutions and firms in life science while other constituencies may have more conservative sectors.

**Table 1:** Citizen and representative acceptance of stem cell research.

	Logit				OLS	
	(1)	(2)	(3)	(4)	(5)	(6)
Representative	-0.1223**	-0.1273***	-0.1082**	-0.1442***	-0.0887**	-0.0994**
	(0.0487)	(0.0454)	(0.0490)	(0.0528)	(0.0383)	(0.0392)
Female	-0.1660***	-0.1623***	-0.1195**	-0.1295***	-0.1040**	-0.0976***
	(0.0504)	(0.0513)	(0.0587)	(0.0485)	(0.0480)	(0.0370)
Age	0.0646	0.1293	0.0853	0.1576	0.0037	0.0058
	(0.1430)	(0.1721)	(0.1872)	(0.2095)	(0.0073)	(0.0067)
Age squared	-0.0328	-0.0959	-0.0770	-0.1447	-3.3e-05	-5.3e-05
	(0.1494)	(0.1714)	(0.1905)	(0.2187)	(7.2e-05)	(6.7e-05)
Married		-0.0188	-0.0323	-0.0220	-0.0345	-0.0244
		(0.0429)	(0.0376)	(0.0499)	(0.0365)	(0.0345)
Divorced		-0.1483*	-0.1302	-0.1578*	-0.1098	-0.1185*
		(0.0878)	(0.0795)	(0.0822)	(0.0675)	(0.0674)
University education		0.0012	0.0295	-0.0036	0.0239	-0.0025
		(0.0277)	(0.0276)	(0.0450)	(0.0245)	(0.0271)
Catholic		-0.0239	-0.0426	-0.0269	-0.0380	-0.0236
		(0.0394)	(0.0461)	(0.0493)	(0.0369)	(0.0340)
Left party support			-0.2362***		-0.2081***	
			(0.0633)		(0.0564)	
Right party support			0.0310		0.0267	
			(0.0532)		(0.0448)	
Social democrats				0.1314		0.1258
				(0.0971)		(0.0835)
Liberals				0.4367***		0.3919***
				(0.0806)		(0.0666)
Right convservative				0.3010***		0.2843***
				(0.0996)		(0.0870)
Greens				-0.1101*		-0.0681
				(0.0606)		(0.0498)
Other smaller party				-0.2081		-0.1956
				(0.1311)		(0.1252)
No party affiliation declared				0.3077***		0.3306***
				(0.0719)		(0.0882)
Constituency fixed effects	YES	YES	YES	YES	YES	YES
R2	0.121	0.127	0.173	0.289	0.123	0.206
Brier	0.197	0.196	0.187	0.169		
n. Obs.	631	631	631	631	631	631

Notes: The dependent variable for all estimations is "Individual votes YES"; that is, acceptance of stem cell research. Estimated robust clustered (cantonal level) standard errors are reported throughout Table 1. Discrete effects, reported for the logit models, represent the estimated change in the probability of an individual voting yes from zero to one (for dummy variables) or from the first to the third quartile. Dummies: politician (with citizen as the reference group), female, married (or in partnership), divorced, university education, Roman Catholic, party support or affiliation (with Center or Christian democrats as the reference group). All estimates include an intercept.

***, **, and * indicate a mean significance level of below 1%, between 1 and 5%, and between 5 and 10%, respectively.

Nevertheless, when citizens and politicians were analysed separately (Table 2, columns 1–3 and 4–6, respectively) with additional factors controlled for, the gender effect was no longer statistically significant for politicians but marginally significant for citizens. For politicians, the number of years on the National Council did not matter, but party affiliation was of notable import. We introduce party affiliations with a dummy coding of categorical variables for parties. The Christian democrats serve as the control group (reference category) and all other mutually exclusive party affiliations are included as dummies. The results show that Liberals and politicians from the Conservative right being more in favour of the liberal bill than Christian democrats. The coefficient of the Social democrats is not statistically different to the reference category controlling for other party affiliations. Green party politicians were opposed to the bill in comparison to the Christian democrats. This result is in direct contrast to a U.S. study showing Republicans as less likely to favour embryonic stem cell research [9]. No other politician characteristics were statistically significant.

**Table 2:** Citizen, representative acceptance of stem cell research and representation of constituency.

	Citizen acceptance only			Representative acceptance only			Pollitician votes as constituency	
	Logit		OLS	Logit		OLS	Logit	OLS
	(1)	(2)	(3)	(4)	(5)	(6)	(7)	(8)
Female	-0.0678**	-0.0907	-0.0769	-0.0411	-0.1285	-0.0465	-0.0478	-0.0329
	(0.0323)	(0.0577)	(0.0497)	(0.1551)	(0.1418)	(0.0935)	(0.1553)	(0.0897)
Age	0.0868	0.1853	0.0066	-0.1960	-0.5344*	-0.0106	-0.2509	-0.0065
	(0.1734)	(0.2297)	(0.0079)	(0.3520)	(0.3034)	(0.0220)	(0.3475)	(0.0219)
Age squared	-0.0830	-0.1790	-6.6e-05	0.2965	0.5918**	1.3e-04	0.3170	9.9e-05
	(0.1696)	(0.2101)	(7.7e-05)	(0.3488)	(0.2714)	(2.1e-04)	(0.3287)	(2.1e-04)
Married	-0.0179	-0.0243	-0.0314	-0.0826	-0.1572	-0.0619	-0.1054	-0.0494
	(0.0403)	(0.0527)	(0.0494)	(0.1585)	(0.1729)	(0.0882)	(0.1675)	(0.0934)
Divorced	-0.0776	-0.1177*	-0.1610*	-0.0733	0.0026	-0.0733	-0.1064	-0.0472
	(0.0564)	(0.0696)	(0.0903)	(0.2239)	(0.2586)	(0.1051)	(0.1986)	(0.1010)
University education	0.0102	-0.0053	-0.0048	0.0105	0.0078	0.0129	-0.0147	0.0072
	(0.0394)	(0.0400)	(0.0386)	(0.1506)	(0.1367)	(0.0695)	(0.1480)	(0.0732)
Catholic	0.0476	0.0387	0.0312	-0.0444	-0.1809*	-0.0694	-0.0568	-0.0222
	(0.0383)	(0.0422)	(0.0315)	(0.1462)	(0.1051)	(0.0499)	(0.1485)	(0.0651)
Social democrats	0.2506***	0.2405**	0.2320***	-0.1480	-0.1737	-0.1514	-0.1324	-0.1434
	(0.0760)	(0.1028)	(0.0888)	(0.1920)	(0.2375)	(0.1778)	(0.2069)	(0.1762)
Liberals	0.5755***	0.5713***	0.4005***	0.4662**	0.5609***	0.4004***	0.5184**	0.4211***
	(0.0849)	(0.0892)	(0.0806)	(0.1903)	(0.1959)	(0.1123)	(0.2048)	(0.1120)
Conservative right	0.1635*	0.1454	0.1716	0.4037***	0.4753***	0.3871***	0.4462***	0.3887***
	(0.0904)	(0.1246)	(0.1123)	(0.1557)	(0.1695)	(0.1269)	(0.1684)	(0.1234)
Greens	-0.0583	-0.0735	-0.1081	-0.4815***	-0.3754*	-0.0858*	-0.4253**	-0.1061*
	(0.0496)	(0.0670)	(0.1021)	(0.1858)	(0.2027)	(0.0518)	(0.2079)	(0.0562)
Other smaller party	0.0241	0.0469	0.0849	-0.3069	-0.3268	-0.3460*	-0.2545	-0.2887
	(0.1359)	(0.2116)	(0.2180)	(0.2071)	(0.2291)	(0.1995)	(0.2225)	(0.2048)
No party affiliation declared	0.1595	0.1443	0.1186					
	(0.1636)	(0.2422)	(0.2035)					
No church attendance		0.2000***	0.1540***					
		(0.0730)	(0.0355)					
Low income		0.0216	0.0138					
		(0.0515)	(0.0425)					
Impact country		0.1613***	0.0458***					
		(0.0421)	(0.0092)					
Number of interest groups					0.0674	0.0060	0.0864	0.0052
					(0.0727)	(0.0046)	(0.0604)	(0.0043)
Active years on National Council					0.0694	0.0022	0.0480	0.0019
					(0.1498)	(0.0077)	(0.1099)	(0.0077)
% Canton yes					0.1908**	1.0903***		
					(0.0773)	(0.2792)		
Constituency fixed effects	YES	YES	YES	NO	NO	NO	NO	NO
R2	0.230	0.333	0.242	0.588	0.646	0.506	0.646	0.478
Brier	0.175	0.155		0.120	0.108		0.108	
n. Obs.	471	471	471	160	160	160	160	160

Notes: The dependent variable for all estimations is "Individual votes YES"; that is, acceptance of stem cell research. Estimated robust clustered (cantonal level) standard errors are reported throughout Table 2. Discrete effects, reported for logit models, represent the estimated change in the probability of an individual voting yes from zero to one (for dummy variables) or from the first to the third quartile. Dummies: politician (with citizen as the reference group), female, married (or in partnership), divorced, university education, Roman Catholic, party affiliation (with Christian democrats as the reference group), no (infrequent) church attendance, and low income (lowest tercile). All estimates include an intercept.

***, **, and * indicate a mean significance level of below 1%, between 1 and 5%, and between 5 and 10%, respectively.

Among voters, church attendance was negatively linked to bill acceptance (Table 2, columns 2 and 3), which increased support by over 15.0 percentage points for those who never or seldom attended church. Religious denomination, however (i.e., Roman Catholic or not), played no statistically significant role, suggesting that it is the church as an institution that is the producer of ideologies [25,26]. For voters party affiliation mattered too with Liberals and Social democrats supporters being 57.1 and 24.1 percentage points more likely in favour of the bill than the reference group (Christian democrats). Again, we employ a standard dummy coding of categorical variables for parties Supporters of the conservative right (the Swiss People’s Party) did not differ statistically from the reference group nor did supporters for the Green party or citizens that did not declare any affiliation to a specific party. The different results for party affiliations for voters and politicians point to deviations between the two groups.

We did observe a moderate match between National Council members’ votes and their cantonal/district outcomes (%YES canton). Citizens who believed that stem cell research is important for Switzerland were also more likely to vote in favour of the bill (on a scale from 1 to 10). In fact, an increase from the first to the third quartile for this variable increased the probability of a yes vote by 16.1 percentage points. We found no difference, however, between low and high income voters. All results remained qualitatively identical when we estimated a multilevel logistic model with random effects for cantons.

To explore factors which explain deviations between voters and politicians we analyze the sample of politicians but change the dependent variable to whether the majority of voters in a constituency voted in line with the politicians representing the constituency in Table 2, columns 7–8. Our results show that party affiliations of politicians are the driving factor that explains deviations of politicians from their constituency’s preferences. Politicians from the Liberal party and the Conservative right have a higher probability to represent the their constituency’s preferences while politicians from the Green party have a lower probability to do so compared to the reference group. This pattern of results is consistent with the effects found when analysing samples of politicians and citizens separately.

Because of the high level of direct democracy in Switzerland, its citizens are generally well informed about upcoming referenda through intense public discourse and official booklets. These latter, which include the exact text of the legislative paragraphs to be modified or introduced into the law or constitution, provide objective information on the referendum issue. Counter-committees that have collected signatures may also provide outlines of their arguments, and parliament itself usually declares its position. Thus, citizens are provided a complete picture not only of the referendum content but also of the different perspectives. The opportunity to vote then encourages citizens to be informed about and discuss the entire issue. According to our findings, in this environment, citizens are more likely than politicians to favour embryonic stem cell research, suggesting that social discussion may help bring about agreement on shared principles, professional norms, and procedural conditions related to stem cell research. Citizen involvement through direct democracy might thus provide a way to bridge polarization in the stem cell debate.

## Conclusions

Citizens care whether scientists are trustworthy, act transparently, and serve the public interest. Even scientists themselves have requested that journal editors and funding agencies adhere to the guidelines of the International Society for Stem Cell Research to encourage compliance [27]. Meanwhile, however, the monitoring function is being taken over by institutional and ethics review boards or committees. Such bodies need to require evaluation of the scientists’ rationale in proposals for embryo-creating research, especially as technical barriers continue to fall because of repeated embryo cloning and stem cell generation [28]. Ultimately, however, research involving embryonic stem cells is likely to remain controversial and dependent on citizen values. Direct involvement of citizens in the decision process and the resulting public discussion on stem cell research may engender beneficial effects and more broadly acceptable policy outcomes.

## Supporting Information

S1 Table Data description and sources. (PDF)

S1 ZIP File Raw data and code. (ZIP)

## References

[1] Blow N. In Search of Common Ground. Nature. 2008; 451: 855–858. 10.1038/451855a 18273022

[2] Williams C, Kitzinger J, Henderson L. Envisaging the Embryo in Stem Cell Research: Rhetorical Strategies and Media Reporting of the Ethical Debates. Sociology of Health & Illness. 2003; 25(7): 793–814. 19774747 10.1046/j.1467-9566.2003.00370.x

[3] Check E. Stem Cells: Candidates Take Opposing Stances on Medical Research. Nature. News blog, published online, 2004 Sept 15,

[4] Check E. US Stem Cell Research Harmed by Uncertainty. Nature, News Blog, 2011 Feb 3, http://blogs.nature.com/news/2011/02/uncertainty_hurting_us_stem_ce.html.

[5] Nisbet MC, Brossard D, Kroepsch A. Framing Science: The Stem Cell Controversy in an Age of Press/Politics. International Journal of Press/Politics. 2003; 8: 36–70.

[6] Levine AD. Policy Uncertainty and the Conduct of Stem Cell Research. Cell Stem Cell. 2011; 8: 132–135. 10.1016/j.stem.2011.01.002 21295270

[7] Moon S, Cho SB. Differential Impact of Science Policy on Subfields of Human Embryonic Stem Cell Research. PLoS One. 2014; 9(4): e86395 10.1371/journal.pone.0086395 24717403 PMC3981698

[8] Vakili K, McGahan AM, Rezaie R, Mitchell W, Daar AS. Progress in Human Embryonic Stem Cell Research in the United States between 2001 and 2010. PLoS ONE. 2015; 10(3): e0120052 10.1371/journal.pone.0120052 25812114 PMC4374681

[9] Nisbet M, Markowitz M. Understanding Public Opinion in Debates over Biomedical Research: Looking beyond Political Partisanship to Focus on Beliefs about Science and Society. PLoS One. 2014; 9(2): e88473 10.1371/journal.pone.0088473 24558393 PMC3928253

[10] Holden C. Scientists Protest ‘Misrepresentation’ As Senate Vote Looms, Science. 2007; 315: 315–316. 10.1126/science.315.5810.315a 17234921

[11] Lovell-Badge R. The Future for Stem Cell Research. Nature. 2001; 414: 88–91. 10.1038/35102150 11689952

[12] Borgelt EL, Dharamsi S, Scott CT. Dear Students: Stem Cell Scientists’ Advice to the Next Generation. Cell Stem Cell. 2013; 12 (June 6): 652–655. 23746974 10.1016/j.stem.2013.05.007

[13] Wadman M. Court Quashes Stem-Cell Lawsuit. Nature. 2011; 476: 14–15. 10.1038/476014a 21814250

[14] Holden C, Vogel G. A Seismic Shift for Stem Cell Research. Science. 2008; 319: 560–563. 10.1126/science.319.5863.560 18239100

[15] Cyranoski D. The Black Box of Reprogramming. Nature. 2014; 516: 162–164. 10.1038/516162a 25503218

[16] Editorial. Nature Medicine. 2013; 19(7): 820. 10.1038/nm.3266 23836223

[17] Dasgupta I, Bollinger J, Mathews DJH, Neumann NM, Rattani A, Sugarman J. Patients’ Attitudes toward the Donation of Biological Materials for the Derivation of Induced Pluripotent Stem Cells. Cell Stem Cell. 2014; 14 (January 2): 9–12. 24388172 10.1016/j.stem.2013.12.006 PMC4733848

[18] Cyranoski D, Reardon S. Embryo Editing Sparks Epic Debate. Nature. 2015; 520: 593–594. 10.1038/520593a 25925450

[19] Kamenova K, Caulfield T. Stem Cell Hype: Media Portrayal of Therapy Translation. Science Translational Medicine. 2015; 7: 278ps4 10.1126/scitranslmed.3010496 25761887

[20] Hayden EC. Hope on the Line. Nature. 2014; 511: 19–21. 10.1038/511019a 24990726

[21] Editorial note. Nature. 2014; 508: 287.

[22] Stadelmann D, Portmann M, Eichenberger R. Quantifying Parliamentary Representation of Constituents’ Preferences with Quasi-Experimental Data. Journal of Comparative Economics. 2013; 41: 170–180.

[23] Stadelmann D, Portmann M, Eichenberger R. Full Transparency of Politicians' Actions Does Not Increase the Quality of Political Representation. Journal of Experimental Political Science. 2014; 1, 16–23.

[24] Hessami Z. How Do Voters React to Complex Choices in a Direct Democracy? Evidence from Switzerland. Kyklos. 2016; 69: 263–293.

[25] Torgler B. The Importance of Faith: Tax Morale and Religiosity. Journal of Economic Behavior & Organization. 2006; 61: 81–109.

[26] Gutmann J. Believe, But Verify? The Effect of Market Structure on Corruption in Religious Organizations. Kyklos. 2015; 68: 153–164.

[27] Daley GQ, Ahrlund-Richter L, Auerbach JM, Benvenisty N, Charo RA, Chen G, et al The ISSCR Guidelines for Human Embryonic Stem Cell Research. Nature. 2007; 315: 603–604. 10.1126/science.1139337 17272706

[28] Hyun I. Regulate Embryos Made for Research. Nature. 2014; 509 (1 May): 27–28. 24784200 10.1038/509027a

