# Supplementary material for: Voting on Embryonic Stem Cell Research: Citizens More Supportive than Politicians
Source: PLoS One. 2017 Jan 26;12(1):e0170656. doi: 10.1371/journal.pone.0170656 (PMC5268364; doi:10.1371/journal.pone.0170656)
Supplement: S1 Table — (PDF) [file pone.0170656.s001.pdf]

S1 Table. Data description and sources.

Variable	*Description and sources*	*Mean*	*SD*
Accept research	Indicator variable: 1 if politcian/citizen voted "yes" for stem cell research. Swiss Parliamentary Services and FORS Vox Survey.	0.686	0.464
Politician	Indicator variable: 1 if politician. Own construction.	0.254	0.435
Female	Indicator variable: 1 if female. Swiss Parliamentary Services and FORS Vox Survey.	0.449	0.498
Age	Age of politican/citizen in years. Swiss Parliamentary Services and FORS Vox Survey.	51.360	14.490
Married	Indicator variable: 1 if married or partnership. Swiss Parliamentary Services and FORS Vox Survey.	0.650	0.477
Divorced	Indicator variable: 1 if divorced or separated. Swiss Parliamentary Services and FORS Vox Survey.	0.079	0.270
University education	Indicator variable: If university education. Swiss Parliamentary Services and FORS Vox Survey.	0.296	0.457
Catholic	Indicator variable: 1 if catholic for citizen or catholic majority in canton for politician. BFS - Federal Office for Statistics and FORS Vox Survey.	0.439	0.497
Left party	Indicator variable: 1 if political affiliation of citizen left leaning (center of no affilation is omitted category) or politician from left party. Swiss Parliamentary Services and FORS Vox Survey.	0.244	0.430
Right party	Indicator variable: 1 if political affiliation of citizen right leaning (center of no affilation is omitted category) or politician from right party. Swiss Parliamentary Services and FORS Vox Survey.	0.208	0.406
Social democrats	Indicator variable: 1 if political affiliation of citizen/politician equals Social democrats (SP). Swiss Parliamentary Services and FORS Vox Survey.	0.241	0.428
Liberals	Indicator variable: 1 if political affiliation of citizen/politician equals Liberals (FDP). Swiss Parliamentary Services and FORS Vox Survey.	0.160	0.367
Conservative right	Indicator variable: 1 if political affiliation of citizen/politician equals Conservative right (SVP). Swiss Parliamentary Services and FORS Vox Survey.	0.136	0.343
Greens	Indicator variable: 1 if political affiliation of citizen/politician equals Green party (GPS) or Green liberal party (GLP). Swiss Parliamentary Services and FORS Vox Survey.	0.089	0.412
Other smaller party	Indicator variable: 1 if political affiliation of citizen/politician does not equal Christian democrats, Social democrats, Liberals, Conservative right or Greens. Swiss Parliamentary Services and FORS Vox Survey.	0.376	0.485
No party affiliation declared	Indicator variable: 1 if citizen declared not party affiliation. Politicians always have a party affilaition. FORS Vox Survey.	0.307	0.462
No church attendance	Indicator variable: 1 if citizen reports no or low church attendance (not available for politicians). FORS Vox Survey.	0.452	0.498
Low income	Indicator variable: 1 if citizen is in low income tercile (not available for politicians). FORS Vox Survey.	0.329	0.470
Impact country	Citizens evaluate referendum to have high (10) or low (1) impact on country (not available for politicians). FORS Vox Survey.	7.391	2.581
Number of interest groups	Number of interes group affiliations of politician (not available for citizens). Swiss Parliamentary Services.	4.275	5.614
Active years on National Council	Number of years in parliament at day of referendum (not available for citizens). Swiss Parliamentary Services.	4.881	4.513
% Canton yes	Yes share at referendum in electzoral district/canton (not relevant for citizens). Swissvotes Database.	0.663	0.080

Notes: Unweighted descriptive statistics for voters and politicians combined. Data sources indicated next to variable descriptions. The raw data and the R code to obtain the main results are provided as supplementary material.
